# Screening for maternal infection in pregnancy to prevent preterm birth: a systematic review

**DOI:** 10.61622/rbgo/2026rbgo56

**Published:** 2026-10-13

**Authors:** Zaenab Azzahra, Fera Ibrahim, Dwiana Ocviyanti

**Affiliations:** 1 Universitas Indonesia Faculty of Medicine Department of Clinical Microbiology Jakarta Indonesia Department of Clinical Microbiology, Faculty of Medicine, Universitas Indonesia, Jakarta, Indonesia; 2 Dr. Cipto Mangunkusumo National Central Public Hospital Department of Clinical Microbiology Jakarta Indonesia Department of Clinical Microbiology, Dr. Cipto Mangunkusumo National Central Public Hospital, Jakarta, Indonesia; 3 Universitas Indonesia Faculty of Medicine Department of Obstetrics and Gynecology Jakarta Indonesia Department of Obstetrics and Gynecology, Faculty of Medicine, Universitas Indonesia, Jakarta, Indonesia; 4 Dr. Cipto Mangunkusumo National Central Public Hospital Department of Obstetrics and Gynecology Jakarta Indonesia Department of Obstetrics and Gynecology, Dr. Cipto Mangunkusumo National Central Public Hospital, Jakarta, Indonesia

**Keywords:** Asymptomatic bacteriuria, Bacterial vaginosis, Pregnancy, Preterm birth, Prenatal screening

## Abstract

**Objective:**

To analyze whether screening for maternal infections during pregnancy to prevent premature birth is associated with a reduction in premature birth.

**Data sources:**

A systematic review was conducted. PubMed, Scopus, Cochrane Library and ProQuest databases were screened for original studies carried out on pregnant women and used Medical Subject Headings (MeSH) terms and free-text keywords related to pregnancy, infection screening, and preterm birth.

**Selection of studies:**

Randomized and observational studies that evaluated antenatal screening for maternal genital or urinary infections, compared screened populations with usual care without additional targeted infection screening, and reported preterm birth (<37 weeks) or related obstetric outcomes were included.

**Data collection:**

Study selection and extraction were performed independently by two reviewers.

**Data synthesis:**

Out of 858 records obtained, 10 studies met the inclusion criteria. These studies screened for various genital and urinary infections. The effect estimates for the unselected antenatal populations were mostly not significant. Nevertheless, maternal infection screening in selected or high-risk populations and subgroup analyses among nulliparous women screened for bacterial vaginosis (BV) or *Chlamydia trachomatis/Neisseria gonorrhoeae* (CT/NG) was associated with reduction in preterm birth. Marked heterogeneity in populations, settings, target infections, and screening protocols limited direct comparison across studies.

**Conclusion:**

Antenatal infection screening in unselected pregnant populations did not show a significant reduction in preterm birth. However, more favourable findings were observed in selected or high-risk populations, although overall effects are heterogeneous. Further well-designed studies in clearly defined target populations are needed to define optimal screening strategies for routine practice.

**PROSPERO registration number**: CRD420251244205

## Introduction

Preterm birth or delivery before the completion of 37 weeks of gestation is one of the main causes of morbidity and mortality of newborns and is one of the main global public health issues which contribute to long-term adverse health outcomes in childhood and adulthood.^(1-3)^ In Indonesia, preterm birth is estimated to occur in about 10–15% of pregnancies, corresponding to roughly 600,000 preterm births annually, and imposes a substantial burden on the national health system. Long-term cohort data from Indonesia underline the need for robust, context-specific evidence to guide strategies for preterm birth prevention and care.^(4)^

Maternal infections are recognised as major contributors to spontaneous preterm birth. It is estimated that 25–40% of preterm deliveries are associated with maternal infections, including intrauterine, lower genital tract, urinary, and systemic infections.^(5,6)^ Microbiological studies of amniotic fluid, fetal membranes, and placental tissues in women delivering preterm have identified a characteristic spectrum of microorganisms. Frequently isolated bacteria *include Ureaplasma species, Mycoplasma hominis,* group B *Streptococcus, Candida spp.,* and *Trichomonas vaginalis*.^(7,8)^

Several infection-targeted interventions have demonstrated beneficial effects on preventing preterm birth outcomes. A scoping review of systematic reviews identified consistent reductions in preterm birth risk with the screening and treatment of asymptomatic bacteriuria (ASB), treatment of syphilis and vaginal candidiasis, and highlighted infection-related interventions as an important group within primary prevention strategies.^(9)^ Other evidence shows that antibiotic treatment of bacterial vaginosis (BV), ASB, and group B *Streptococcus* colonisation can reduce preterm birth in selected populations.^(5)^ Furthermore, a recent systematic review of clinical practice guidelines on prediction and prevention of spontaneous preterm birth found broad consensus on universal screening and treatment for ASB, and on screening and treating BV in high-risk pregnancies as part of preterm birth prevention bundles.^(10)^

Currently, available evidence regarding antenatal infection screening is limited to single pathogens or specific diseases. A review of current studies evaluating integrated screening and management strategies for BV, sexually transmitted infections, and urinary tract infections (UTI) in routine prenatal care is needed. Therefore, this systematic review aims to assess whether such screening and management programs during pregnancy, compared with standard prenatal care without additional targeted screening, are associated with a reduced incidence of preterm birth.

## Methods

### Search protocol and study selection

This systematic review was conducted in accordance with the Preferred Reporting Items for Systematic Reviews and Meta-Analyses (PRISMA) guidelines and the protocol was registered in PROSPERO (ID: CRD420251244205).^(11)^ The research question was formulated using the Patient–Intervention–Comparison–Outcome (PICO) framework as follows: Population (P): pregnant women at any gestational age. Intervention (I): any screening strategy for maternal infection during pregnancy using clinical, microbiological, molecular or point-of-care tests as part of antenatal care. Comparison (C): no screening or usual care without additional targeted infection screening. Outcomes (O): preterm birth (<37 completed weeks of gestation).

Original research articles evaluating the effect of infection screening during pregnancy on preterm birth were identified across four databases, including PubMed, Scopus, ProQuest, and Cochrane. No restriction on publication year was applied. The literature search was conducted in April 2026. A combination of Medical Subject Headings (MeSH) terms and free-text keywords was used, adapted to each database. The core search strategy included terms related to pregnancy, infection, screening, and preterm birth: ("pregnancy" OR "pregnant women") AND ("infection screening" OR "infection screening test" OR "antenatal screening") AND ("preterm birth" OR "preterm labour" OR "premature birth"). The complete search strategy for each database is provided in Supplementary Material - Table 1S.

The titles and abstracts retrieved from the search were imported into a Microsoft Excel spreadsheet and screened for duplicates and eligibility. Two reviewers independently screened titles and abstracts to identify potentially relevant studies. Full-text articles were then obtained for all records considered potentially eligible and assessed in detail against the inclusion and exclusion criteria. Discrepancies between reviewers were resolved through discussion or consultation with a third reviewer when necessary.

### Eligibility criteria

There was no limitation on geographic region or health-care setting. The search was restricted to studies published in English. Inclusion criteria were: (1) original clinical studies conducted in humans; (2) pregnant women as the study population; (3) evaluation of a defined antenatal screening strategy for maternal genital or urinary infection, including laboratory-based, molecular, point-of-care, or standardized clinical screening approaches; (4) studies that compared screened versus unscreened populations, or usual antenatal care without additional targeted infection screening; (5) studies that reported outcomes related to preterm birth (<37 weeks) or closely related obstetric outcomes. Exclusion criteria were: (1) systematic, narrative, or literature reviews, study protocol, conference abstracts, editorials and case reports; (2) studies in non-pregnant populations or in animal models; (3) studies focusing solely on treatment of infection after diagnosis without evaluating the impact of a defined screening program or strategy.

### Study selection and data extraction

For each study meeting the inclusion criteria, data were extracted including first author, year of publication, and country, study design, and characteristics of the study population, type of infection targeted by screening (e.g., BV, UTI, sexually transmitted infections, or other infections), outcomes assessed, and effect estimates (risk ratios, odds ratios). For descriptive purposes, individual study effect sizes (odds ratios and risk ratios with 95% confidence intervals) were displayed in a forest plot, but no pooled summary estimate was calculated.

### Study quality appraisal

The methodological quality and risk of bias of the included studies were assessed using the Mixed Methods Appraisal Tool (MMAT).^(12)^ Each study was evaluated across key domains, including selection of participants, measurement of exposure and outcomes, comparability of groups, control of confounding factors, and completeness of follow-up. Disagreements in quality ratings were resolved by consensus or consultation with a third reviewer. The results of study quality appraisal were used to interpret the findings rather than to generate an overall quantitative quality score.

### Data synthesis

Data were synthesized narratively according to study design, study population, target infection, screening strategy, and reported outcomes. Study-level effect estimates for preterm birth were extracted and summarized descriptively. Individual study effect estimates were displayed in a forest plot for descriptive purposes only. Meta-analysis was not performed, and no pooled effect estimate was calculated.

## Results

### Study selection

A total of 858 records were identified through the database search (PubMed = 562, Scopus = 149, ProQuest = 129, Cochrane = 18). After removal of 237 duplicates, 621 studies remained for title and abstract screening. Of these, 601 studies were excluded because they were not relevant to the review question. The full texts of the remaining 20 articles were assessed for eligibility, leading to the exclusion of 10 studies that did not meet the predefined inclusion and exclusion criteria. Ultimately, 10 studies fulfilled all criteria and were included in the qualitative synthesis of this systematic review. The flow chart of study selection is presented in figure 1.

**Figure 1:**
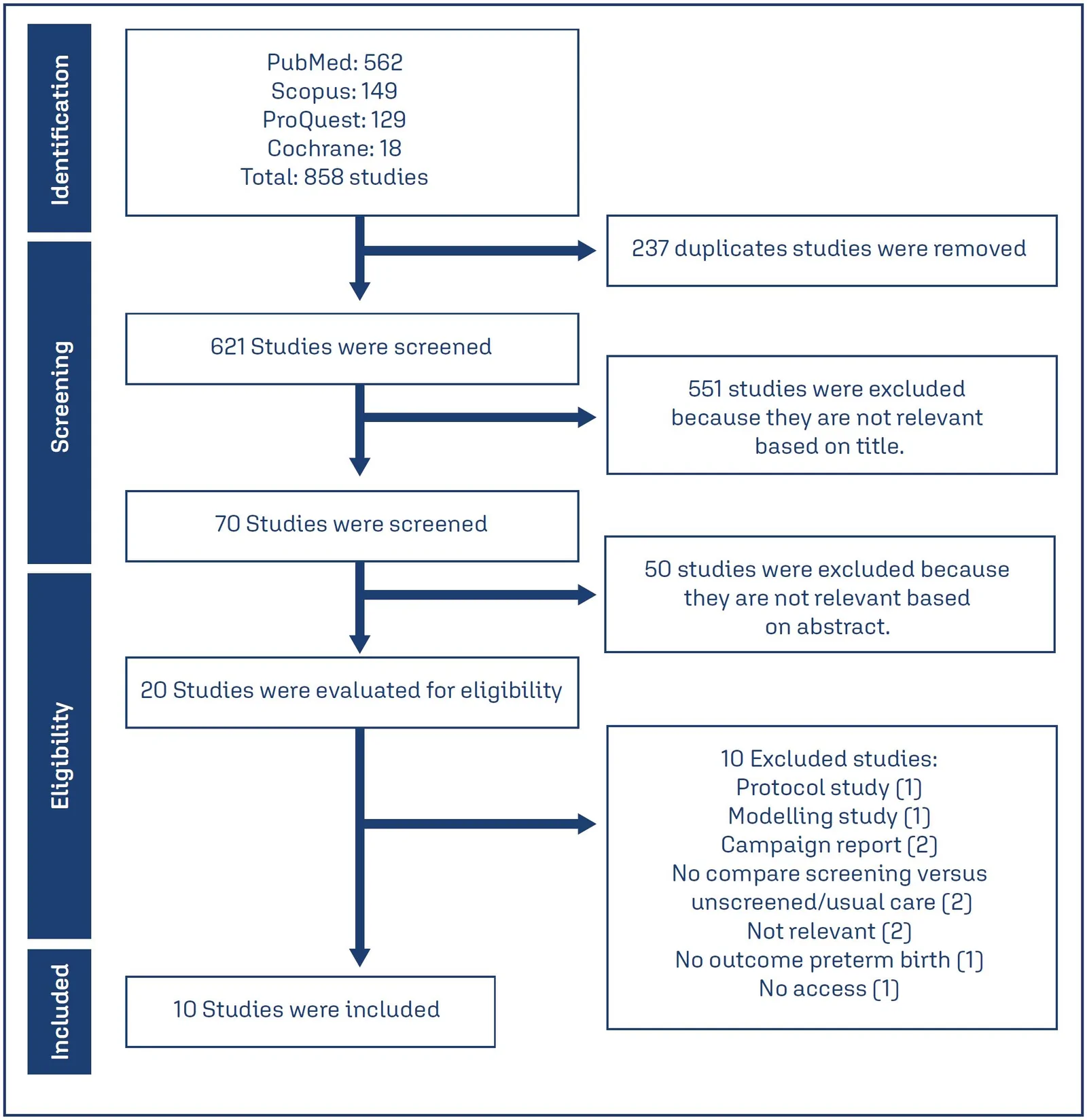
Flowchart of the study selection process

### Methodological quality of included studies

Study quality was evaluated using the MMAT, which is shown in chart 1. Among the four randomized controlled trials, the number of MMAT criteria rated "Yes" ranged from three to five. For the six quantitative non-randomized studies, between three and five MMAT criteria were rated "Yes". Overall, the included studies were considered to be of moderate methodological quality.

**Chart 1:** Methodological quality assessment of included studies using the MMAT

Design study and studies	Assessment Criteria					Number of criteria rated "Yes"
**Randomized controlled trials**	**Is randomization appropriately performed**	**Are the groups comparable at baseline?**	**Are there complete outcome data?**	**Are outcome assessors blinded to the intervention provided?**	**Did the participants adhere to the assigned intervention?**	
Riddell et al., 2024^(13)^	Yes	Can't tell	Yes	Yes	Can't tell	3
Bretelle et al., 2023^(14)^	Yes	Yes	Yes	No	Yes	4
Gehani et al., 2021^(15)^	Yes	Yes	Yes	Yes	Yes	5
Lee et al., 2019^(16)^	Yes	Yes	Yes	No	Can't tell	3
**Quantitative non-randomized**	**Are the participants representative of the target population?**	**Are measurements appropriate regarding both the outcome and intervention (or exposure)?**	**Are there complete outcome data?**	**Are the confounders accounted for in the design and analysis?**	**During the study period, is the intervention administered (or exposure occurred) as intended?**	
Wynn et al., 2024^(17)^	Yes	Yes	Yes	Yes	Yes	5
Roero et al., 2024 ^(18)^	Yes	Yes	Yes	Can't tell	Yes	4
Houlihan et al., 2023^(19)^	Yes	Yes	Yes	No	Can't tell	3
Toboso Silgo et al., 2021^(7)^	Yes	Yes	Yes	No	Yes	4
Kiss et al., 2010^(20)^	Yes	Yes	Yes	No	Yes	4
Farr et al., 2015^(21)^	Yes	Yes	Yes	Yes	Yes	5

MMAT: Mixed Methods Appraisal Tool. MMAT results are presented for descriptive purposes only. No overall quality score was calculated

### Characteristics of included studies

The characteristics and main findings of the studies are summarised in chart 2. The ten included studies were published between 2010 and 2024 and comprised four randomized or cluster-randomized trials, six observational studies. Study sites spanned low-, middle-, and high-income settings, including Botswana, Papua New Guinea, India, France, Spain, Ireland, Italy, Bangladesh, and Austria. The screening strategies evaluated targeted a range of genital and urinary infections, including bacterial vaginosis, asymptomatic bacteriuria, *Chlamydia trachomatis/Neisseria gonorrhoeae*, mixed genitourinary infections, and abnormal vaginal flora. Overall, the studies reported effect estimates for preterm birth and related outcomes using odds ratios (OR), adjusted odds ratios (aOR), adjusted risk ratios (aRR), and risk ratios (RR). Across unselected antenatal populations, most studies did not demonstrate a statistically significant reduction in preterm birth associated with routine infection screening. More favourable findings were reported in selected or high-risk populations and in within-study subgroup analyses, including nulliparous women screened for bacterial vaginosis or *Chlamydia trachomatis/Neisseria gonorrhoeae*, and among women with confirmed infection who received targeted treatment.

**Chart 2:** Characteristics and main findings of studies evaluating maternal infection screening and preterm birth

Authors	Study design	Location	Patients	Screening test	Findings / statistical test results	Level of evidence
Wynn et al., 2024^(17)^	Non-randomized, cluster-controlled, crossover trial	Gaborone, Botswana	Pregnant women ≥15 years, first antenatal visit, gestational age ≤27 weeks, asymptomatic for urogenital infection; 436 pregnancies included.	Vaginal swab using GeneXpert® CT/NG (NAAT)	Primary composite outcome (preterm birth or low birth weight): aOR 0.59 (95% CI 0.28-1.24). Preterm birth: aOR 0.59 (0.25-1.38), aRR 0.65; 95% CI 0.33-1.28 In nulliparous subgroup: composite outcome aOR 0.20 (95% CI 0.07-0.64), preterm birth aOR 0.22 (95% CI 0.07-0.72), low birthweight aOR 0.23 (95% CI 0.06-0.83).	2b
Roero et al., 2024^(18)^	Retrospective cohort study comparing two screening strategies	Turin, Italy	Asymptomatic women with twin pregnancy; 476 pregnancies included.	Cervicovaginal swab (endocervix and posterior fornix); single vs double testing strategy Single test group (STG): test on 20-22 week only vs Double test group (DTG): 12-14 week and 20-22 week	Double testing had a lower incidence of pPROM: 23.1% in STG vs. 14.4% in DTG (p=0.021). In multivariate analysis, double testing was associated with a decreased risk of pPROM (OR 0.55; 95% CI 0.33–0.93; p=0.025). But there was no significant difference for preterm birth at ≤28, ≤31, or ≤34 weeks. OR 0.97 (0.63-1.48)	2b
Riddell et al., 2024^(13)^	Pragmatic cluster-randomized crossover trial	Papua New Guinea	Pregnant women ≥16 years, antenatal visit at ≤26 weeks’ gestation; 4,526 women included.	Point-of-care testing for *C. trachomatis, N. gonorrhoeae, T. vaginalis*, and BV vs standard syndromic care.	Primary composite outcome (preterm birth <37 weeks, and/or low birthweight <2500 g) RR 1.06 (95% CI 0.78-1.42; p=0.67). Preterm birth only RR 1.07 (95% CI 0.79-1.45; p=0.61). In women with *N. gonorrhoeae* at enrolment, the primary outcome was reduced in the intervention group: RR 0.47 (95% CI 0.25-0.88).	1b
Houlihan et al., 2023^(19)^	Retrospective comparative cohort study	Dublin, Ireland	All women delivering >500 g over 2018-2020 in both hospitals; total 47,676 deliveries.	Routine ASB screening with midstream urine culture vs no screening	No significant difference in antenatal pyelonephritis: 0.35% vs. 0.30% of all births (p=0.34). No difference in preterm birth: (7.3% vs. 7.0%; p=0.21).	2b
Bretelle et al., 2023^(14)^	Multileft randomized, controlled trial (AuTop RCT)	France	Low-risk pregnant women ≥18 years old, ≤ 20 weeks; 6671 pregnancies included.	Systematic molecular screening BV using quantitative real-time PCR	No significant difference in preterm birth <37 weeks: RR 0.83 (95% CI 0.66-1.05; p=0.12). In nulliparous women RR 0.62 (95% CI 0.45-0.84; p interaction =0.003).	1b
Toboso Silgo L et al., 2021^(7)^	Prospective observational cohort study	Madrid, Spain	Pregnant women >18 years, singleton pregnancies; 381 women with complete outcome data included.	First trimester vaginal and endocervical screening	Abnormal vaginal flora increased risk of pPROM approximately 5-fold: OR 5.11 (95% CI 1.20-21.71; p=0.028). No significant difference for preterm delivery (6.1% vs. 6.3%; p=0.938). There were no significant differences in low birth weight or neonatal morbidity.	2b
Gehani et al., 2021^(15)^	Pragmatic randomized controlled trial	India	Pregnant women (13–34 weeks of pregnancy), asymptomatic for UTI; 213 women included.	Rapid point-of-care ASB screening vs usual care	Composite outcome: preterm birth and/or low birth weight: RR 0.64 (95% CI 0.43-0.95; p=0.023). PTB alone: RR 0.76 (95% CI 0.50-1.15); for low birth weight alone: RR 0.38 (95% CI 0.16-0.92).	1b
Lee et al., 2019^(16)^	Unblinded cluster-randomized clinical trial	Sylhet, Bangladesh	Pregnant women/girls aged 15–49 years identified before 19 weeks’ gestation in rural community clusters; 9712 pregnancies enrolled	Home-based screening for abnormal vaginal flora (self-collected vaginal swab, Gram stain, Nugent score ≥4) and urine culture.	Preterm livebirth <37 weeks did not differ significantly between intervention and control groups: 21.8% vs 20.6%; RR 1.07 (95% CI 0.91–1.24). Preterm livebirth <34 weeks: RR 1.00 (95% CI 0.81–1.24).	1b
Farr et al., 2015^(21)^	Retrospective cohort study	Vienna, Austria	Women with singleton high-risk pregnancies delivering between 2005 and 2015; 20,052 pregnancies included.	Gram stain screening for BV, Candida, Trichomonas + treatment	Preterm birth reduced: 9.7% vs 22.3%; aOR 0.38 (95% CI 0.35–0.42)	2b
Kiss et al., 2010^(20)^	Retrospective cohort study	Vienna, Austria	Singleton pregnancies, GA 11–24 weeks; 2986 pregnancies included	Gram stain screening for BV, Candida, Trichomonas + treatment	Preterm birth reduced: 8.2% vs 12.1% (p<0.0001). Very preterm deliveries before 33 weeks were reduced: 1.9% vs 5.4% (p < 0.0001).	2b

* Based on the Level of Evidence issued by the Oxford Left for Evidence-Based Medicine 2011.^(22)^ ASB, asymptomatic bacteriuria; BV, bacterial vaginosis; CI, confidence interval; CT/NG, *Chlamydia trachomatis/Neisseria gonorrhoeae*; NAAT, nucleic acid amplification test; pPROM, preterm premature rupture of membranes; RR, risk ratio; aOR, adjusted odds ratio; aRR, adjusted risk ratio; SOC, standard of care

### Effect estimates for preterm birth

Chart 3 summarises the study-level effect estimate for preterm birth associated with maternal infection screening according to overall study populations, selected/high risk populations, and within-study subgroup analyses. In studies conducted in unselected antenatal populations, effect estimates for preterm birth range from aOR 0.59 (95% CI 0.25-1.38) to an RR of 1.07 (95% CI 0.91-1.24), whereas studies conducted in selected/high-risk populations reported more favourable estimates, including aOR 0.38 (95% CI 0.35-0.42). Subgroup analyses (nulliparous women or women with specific infections) showed effect estimates between 0.22 and 0.62.

**Chart 3:** Effect estimates for maternal infection screening and risk of preterm birth

Study (Unselected antenatal populations)	Population / Screening	Measure	Effect	95% CI
Wynn 2024^(17)^	CT/NG NAAT screening and treatment vs standard of care	aOR	0.59	0.25-1.38
Wynn 2024^(17)^	CT/NG NAAT screening plus treatment vs standard of care	aRR	0.65	0.33-1.28
Riddell 2024^(13)^	Point-of-care testing and treatment vs standard syndromic care	RR	1.07	0.79-1.45
Bretelle 2023^(14)^	Systematic molecular BV screening + treatment vs usual care – PTB <37 weeks	RR	0.83	0.66-1.05
Gehani 2021^(15)^	Rapid point-of-care ASB screening and treatment vs usual care	RR	0.76	0.50-1.15
Lee 2019^(16)^	Screening and treatment of genitourinary infections (UTI + vaginal infections) vs standard care	RR	1.07	0.91–1.24
**Study (Selected/high-risk populations)**	**Population / Screening**	**Measure**	**Effect**	**95% CI**
Roero 2024^(18)^ (twin pregnancy)	Single vs double cervicovaginal testing strategy in twin pregnancy	OR	0.97	0.63-1.48
Farr 2015^(21)^ (high risk pregnancy)	Antenatal screening and treatment of asymptomatic vaginal infections (Gram stain) at 10–16 weeks vs no screening	aOR	0.38	0.35-0.42
**Study (within-study subgroup analyses)**	**Population / Screening**	**Measure**	**Effect**	**95% CI**
Wynn 2024^(17)^ (nulliparous)	CT/NG NAAT screening plus treatment vs standard of care	aOR	0.22	0.07-0.72
Bretelle 2023^(14)^ (nulliparous)	Systematic molecular BV screening and treatment vs usual care	RR	0.62	0.45-0.84
Riddell 2024^(13)^ (positive *N. gonorrhea*)	Point-of-care testing and treatment vs standard syndromic care	RR	0.47	0.25-0.88

ASB, asymptomatic bacteriuria; BV, bacterial vaginosis; CI, confidence interval; CT/NG, *Chlamydia trachomatis/Neisseria gonorrhoeae*; NAAT, nucleic acid amplification test; PTB, preterm birth; RR, risk ratio; OR, odds ratio; aOR, adjusted odds ratio; aRR, adjusted risk ratio

### Synthesis of results

The findings across included studies were heterogeneous in terms of study design, baseline population, type of infection screened, screening method, and reported outcomes. Randomized trials conducted in unselected antenatal populations generally did not demonstrate a significant reduction in preterm birth. Among observational studies, screening and treatment of maternal infections were associated with reduced preterm birth in selected and/or high-risk populations. In addition, subgroup analyses among nulliparous women and women with microbiological *Neisseria gonorrhoea* confirmation at enrolment showed lower effect estimates for preterm birth. Because the included studies differed in reported effect measures (RR, OR, aRR, and aOR), study design, and screening method, a formal meta-analysis was not undertaken, and no pooled effect size was reported. Instead, study-level effect estimates for preterm birth and related outcomes are presented narratively and illustrated in a forest plot for visual comparison only. Figure 2 presents a forest plot of these study-level estimates.

**Figure 2:**
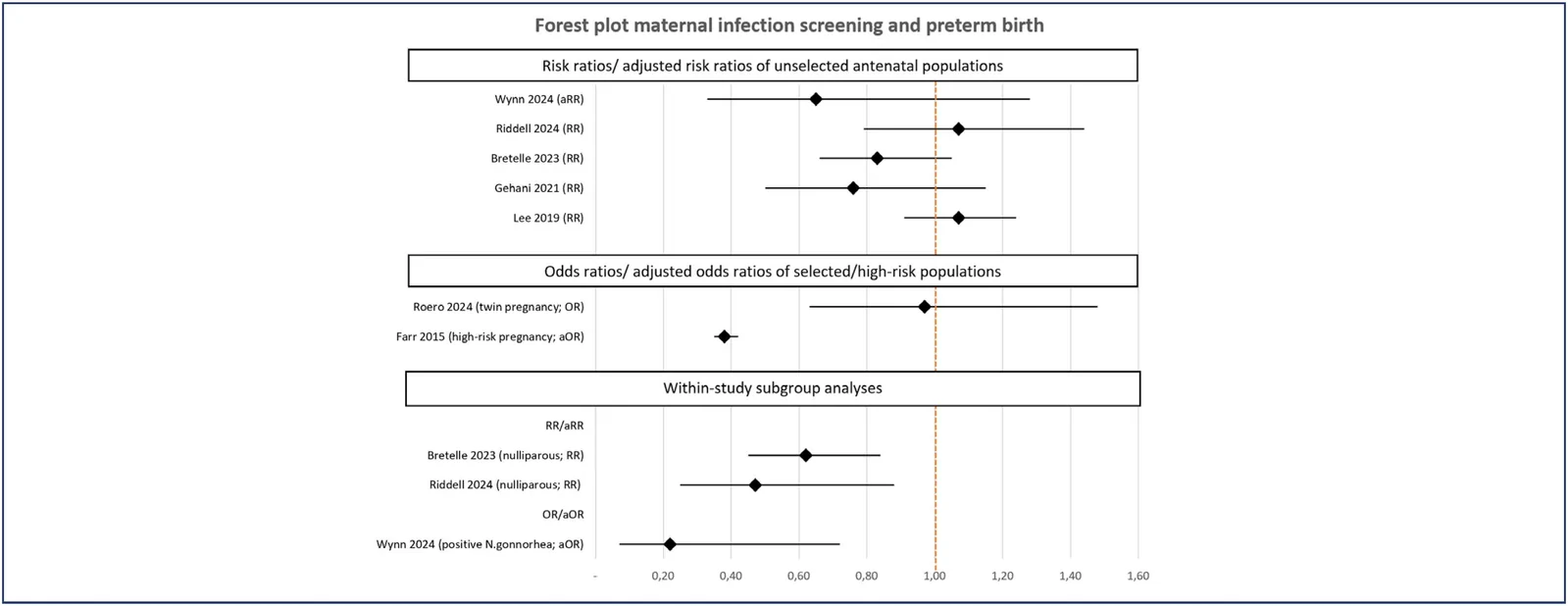
Descriptive forest plot of study-level effect estimates for preterm birth associated with maternal infection screening

Effect estimates are grouped according to study population and effect measure and are presented for visual comparison only. When multiple effect measures were reported for the same study population, one principal estimate was selected. No pooled summary estimate was calculated because of heterogeneity between studies

## Discussion

The association between antenatal screening for maternal infections and preterm birth varied across studies. In unselected antenatal populations, most studies showed no significant reduction in preterm birth. However, more favourable effect estimates were observed among selected/high-risk populations and in within-study subgroup analyses, particularly in nulliparous women and women with confirmed infection at baseline. These findings suggest that the effectiveness of infection screening may depend on the underlying population risk.

### Screening for *Chlamydia trachomatis* and *Neisseria gonorrhoeae* infection

The studies by Wynn et al. and Riddell et al. showed that routine NAAT-based screening for CT/NG among all pregnant women may have only a small impact on prevention of preterm birth at the general population level, however it may still have clinically important benefits in certain high-risk sub-populations. Wynn et al. suggested that the benefits of CT/NG screening may be concentrated in first-time pregnant women or those from settings with a high underlying prevalence of sexually transmitted infections.^(17)^ Riddell et al. found no overall improvement with the combined point-of-care strategy as compared to syndromic management. However, women with *N. gonorrhoeae* at enrolment who were screened and treated had much better outcomes, highlighting the importance of targeting high-risk infections rather than using broad, unselective testing.^(13)^ Adachi et al. conducted a review, analysing 15 studies evaluating the association between antenatal CT screening and treatment during pregnancy and neonatal outcomes. Most studies (13 of 15) reported that identification and treatment of CT infection during pregnancy was associated with a reduced incidence of preterm birth.^(23)^

### Screening for bacterial vaginosis and abnormal vaginal flora

In the cohort study conducted by Toboso Silgo et al. first-trimester screening showed that abnormal flora was strongly associated with preterm premature rupture of membranes (pPROM) but not with overall preterm delivery or neonatal morbidity.^(7)^ Findings from randomized controlled trial by Bretelle et al. suggest that routine molecular screening and treatment for BV in a generally low-risk population may not substantially reduce overall preterm birth.^(14)^ This pattern is consistent with a meta-analyses-study suggesting only modest and uncertain benefit of routine abnormal vaginal flora screening, especially in low-risk populations.^(24)^ In contrast, a study by Kiss et al. found that integration of a simple antenatal screen-and-treat programme for common asymptomatic vaginal infections into routine care was associated with a lower rate of preterm birth in a general population of singleton pregnancies.^(20)^ Nevertheless, this study was retrospective and should therefore be interpreted with caution, particularly in view of potential selection bias and confounding.

In twin pregnancies, the study by Roero et al. demonstrated that a double-test strategy with repeated cervicovaginal swabs in the first and second trimesters significantly reduced pPROM.^(18)^ The favourable estimate also reported by Farr et al. further supports the observation that benefits of infection screening may be more apparent in high-risk pregnancy than in unselected antenatal populations. In that retrospective cohort, integration of a routine screen-and-treat program for asymptomatic vaginal infections in early pregnancy was associated with a substantial reduction in preterm birth. Nonetheless, the non-randomized design and the high-risk tertiary care setting limit direct extrapolation of these findings to universal screening strategies.^(21)^ These results align more closely with a recent systematic review focused on high-risk women, in which screening and treatment of BV reduced recurrent preterm delivery (pooled RR 0.65, 95% CI 0.44–0.98).^(25)^ Additional indirect evidence from Koumans et al. also reported that early identification and treatment of BV/abnormal flora was associated with lower preterm birth, however, this study compared treated and untreated women within an already screened population rather than screened and unscreened groups.^(26)^ These data support a risk-stratified approach in which BV screening is prioritised for women at increased risk of preterm birth, such as nulliparous women or those with a history of preterm delivery, rather than universally applied to all pregnancies.

### Screening for asymptomatic bacteriuria

In the large retrospective comparison of two Irish maternity hospitals conducted by Houlihan et al., introducing routine midstream urine culture at 12–16 weeks did not significantly affect rates of antenatal pyelonephritis or preterm birth compared with no ASB screening, suggesting that in a high-resource setting with good background care, universal culture-based screening may add little benefit for these outcomes.^(19)^ In comparison, the Indian randomized trial showed that a rapid point-of-care ASB test with susceptibility reporting, combined with treatment, was associated with reduced risk of the composite outcome of preterm birth and/or low birth weight.^(15)^

International consensus statements still recommend at least one early-pregnancy urine culture for ASB, primarily to prevent pyelonephritis and its serious maternal complications.^(27)^ A recent systematic review of studies since 2005 found that untreated ASB is clearly associated with an increased risk of pyelonephritis and possibly PROM and low birth weight, but reported inconsistent associations with preterm birth and highlighted substantial methodological limitations in the available data.^(28)^

### Clinical implications

The study in Bangladesh showed that a population-based antenatal screening and treatment programme for abnormal vaginal flora and urinary tract infections did not reduce preterm livebirth overall. It highlighted an important practical issue for low-resource settings: even when screening is implemented at population level, the effectiveness of the programme may be limited by incomplete treatment success, possible antimicrobial resistance, and variation in the local microbiology of abnormal vaginal flora and urinary tract infection.^(16)^ In settings where laboratory resources are limited, maternal infection screening should also be considered carefully to prevent unnecessary antimicrobial exposure that may contribute to overtreatment and antimicrobial resistance.^(29)^

### Strengths and limitations

This review has several strengths. It considered multiple maternal infection screening strategies within a single synthesis, included studies from diverse geographic and economic settings, and examined both randomized and observational evidence. However, the main limitation of this review is the substantial heterogeneity in study design, populations, infection type, method of screening, interventions and outcome measure. Future studies should focus on clearly defined target populations, standardized screening pathways, and comparable outcome measures to determine which strategies are most beneficial for routine antenatal practice.

## Conclusion

Screening for maternal genital and urinary tract infections in unselected populations did not show a significant reduction in preterm birth rates. In contrast, infection screening in selected or high-risk populations and in specific subgroup analyses has been associated with lower preterm birth rates. These findings suggest that targeted infection screening can be considered as part of a comprehensive strategy to prevent preterm birth. Future targeted populations research with standardized screening protocols and outcome measures is necessary to identify infection screening strategies that are effective and feasible for routine antenatal care.

## Data Availability

The research data are described in the article presented.
