# Supplementary material for: Screening for maternal infection in pregnancy to prevent preterm birth: a systematic review
Source: Rev Bras Ginecol Obstet. 2026 Oct 13;48:e-rbgo56. doi: 10.61622/rbgo/2026rbgo56 (PMC13690395; doi:10.61622/rbgo/2026rbgo56)
Supplement: Supplementary file 1 [file 1806-9339-rbgo-48-e-rbgo56-Supp01.pdf]

Supplementary material

Table 1S. Full search strategies and results by database
The database search was conducted in April 2026. No restriction on publication year was applied.

Database	Search strategy	Result
PubMed/ MEDLINE	(("Pregnancy"[Mesh] OR pregnan*[tiab] OR "pregnant women"[tiab] OR antenatal[tiab] OR prenatal[tiab])<br>AND<br>("Infection"[Mesh] OR infection*[tiab] OR "maternal infection"[tiab] OR "genital infection"[tiab] OR "urinary tract infection"[tiab] OR UTI[tiab] OR "bacterial vaginosis"[tiab] OR BV[tiab] OR "sexually transmitted infection"[tiab] OR STI[tiab] OR chlamydia[tiab] OR gonorrhea[tiab])<br>AND<br>("Mass Screening"[Mesh] OR screening[tiab] OR "screening program"[tiab] OR "screening strategy"[tiab] OR testing[tiab] OR "point-of-care testing"[tiab])<br>AND<br>("Premature Birth"[Mesh] OR "preterm birth"[tiab] OR "preterm labour"[tiab] OR "premature birth"[tiab]))<br>Filter: English; Humans	562
Scopus	(TITLE-ABS-KEY(pregnan* OR "pregnant women" OR antenatal OR prenatal)) AND (TITLE-ABS-KEY(infection* OR "maternal infection" OR "genital infection" OR "urinary tract infection" OR UTI OR "bacterial vaginosis" OR BV OR "sexually transmitted infection" OR STI OR "chlamydia" OR "gonorrhea")) AND (TITLE-ABS-KEY(screening OR "screening program" OR "screening strategy" OR testing OR "point-of-care testing")) AND (TITLE-ABS-KEY("preterm birth" OR "preterm labour" OR "premature birth")) AND (LIMIT-TO (DOCTYPE,"ar")) AND (LIMIT-TO (LANGUAGE,"English"))	149
ProQuest	(ab(pregnan* OR "pregnant women" OR antenatal OR prenatal) OR ti(pregnan* OR "pregnant women" OR antenatal OR prenatal))<br>AND<br>(ab(infection* OR "maternal infection" OR "genital infection" OR "urinary tract infection" OR UTI OR "bacterial vaginosis" OR BV OR "sexually transmitted infection" OR STI OR chlamydia OR gonorrhea)<br>OR ti(infection* OR "maternal infection" OR "genital infection" OR "urinary tract infection" OR UTI OR "bacterial vaginosis" OR BV OR "sexually transmitted infection" OR STI OR chlamydia OR gonorrhea))<br>AND<br>(ab(screening OR "screening program" OR "screening strategy" OR testing OR "point-of-care testing")<br>OR ti(screening OR "screening program" OR "screening strategy" OR testing OR "point-of-care testing"))<br>AND<br>(ab("preterm birth" OR "preterm labour" OR "premature birth")<br>OR ti("preterm birth" OR "preterm labour" OR "premature birth"))	129
Cochrane Library	(pregnan* OR "pregnant women" OR antenatal OR prenatal) in Title Abstract Keyword<br>AND<br>(infection* OR "maternal infection" OR "genital infection" OR "urinary tract infection" OR UTI OR "bacterial vaginosis" OR BV OR "sexually transmitted infection" OR STI OR chlamydia OR gonorrhea) in Title Abstract Keyword<br>AND<br>(screening OR "screening program" OR "screening strategy" OR testing OR "point-of-care testing") in Title Abstract Keyword<br>AND<br>("preterm birth" OR "preterm labour" OR "premature birth") in Title Abstract Keyword	18

Searches were limited to English-language studies. Database-specific filters were applied where available.
